# Successful enucleation of large multinodular/plexiform schwannoma of the foot and ankle

**DOI:** 10.1186/s40064-015-1087-3

**Published:** 2015-06-17

**Authors:** Jun Nishio, Shun Mori, Kazuki Nabeshima, Masatoshi Naito

**Affiliations:** Department of Orthopaedic Surgery, Faculty of Medicine, Fukuoka University, 7-45-1 Nanakuma, Jonan-ku, Fukuoka, 814-0180 Japan; Department of Pathology, Faculty of Medicine, Fukuoka University, 7-45-1 Nanakuma, Jonan-ku, Fukuoka, 814-0180 Japan

**Keywords:** Multinodular/plexiform schwannoma, Tibial nerve, Enucleation, MRI, Foot, Ankle

## Abstract

**Background:**

It is often challenging to completely resect multinodular/plexiform schwannomas involving important deep nerves using minimally invasive surgically techniques.

**Case description:**

A 32-year-old woman presented with a 5-year history of a slowly growing, painful mass in the medial aspect of the right ankle. Magnetic resonance imaging (MRI) demonstrated multiple nodular lesions with iso-signal intensity relative to skeletal muscle on T1-weighted sequences and heterogeneous high signal intensity on T2-weighted sequences. Mild to moderate enhancement was identified after gadolinium administration. All 58 tumors were completely enucleated using an intracapsular technique. Histological examination confirmed the diagnosis of schwannoma consisting mainly of Antoni A areas. The burning sensation was relieved immediately after surgery. The patient had no aggravated neurological deficit and was very satisfied with the outcome of the treatment at final follow-up.

**Discussion and evaluation:**

We experienced a very rare case of a large multinodular/plexiform schwannoma arising from the posterior tibial nerve and its larger terminal branch. Our case had the characteristic MRI features of this condition. It is extremely important to differentiate multinodular/plexiform schwannoma from plexiform neurofibroma and malignant peripheral nerve sheath tumor, with complete surgical enucleation being curative.

**Conclusions:**

MRI is a clinically useful modality in the evaluation and detection of deep-seated multinodular/plexiform schwannoma. Intracapsular enucleation seems to be an acceptable treatment for this peculiar tumor located in the foot and ankle.

## Background

Multinodular/plexiform schwannoma, first described in by Harkin et al (1978), is an extremely rare benign neurogenic tumor, usually affecting skin or subcutaneous tissue. It is generally accepted that there is a weak association with neurofibromatosis type 2 (NF2). The lesion is usually slow-growing and rarely exceed 2 cm in greatest diameter (Ikushima et al. 1999). Unlike plexiform neurofibroma, malignant transformation has not been reported (Mohammed et al. 2014). Herein, we describe an unusual case of a deep-seated multinodular/plexiform schwannoma originating from the posterior tibial nerve and its larger terminal branch, which was completely enucleated using an intracapsular technique. To the best of our knowledge, this is the largest multinodular/plexiform schwannoma in the foot and ankle.

## Case description

**Figure 1:**
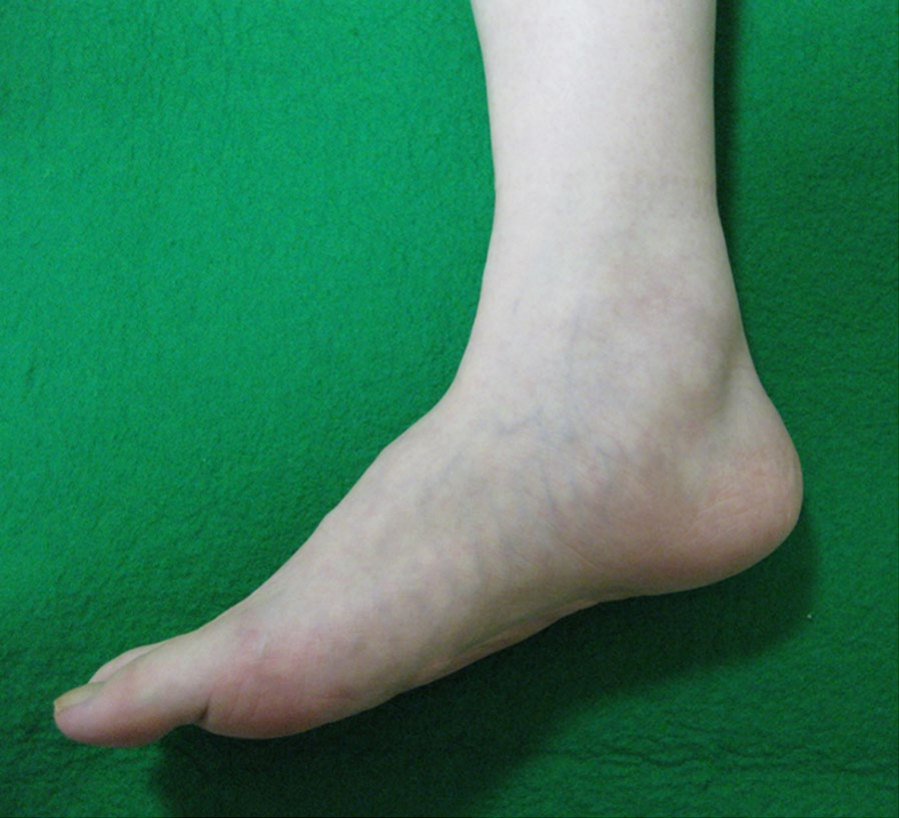
Clinical photograph showing a soft tissue mass in the medial aspect of the right ankle.

**Figure 2:**
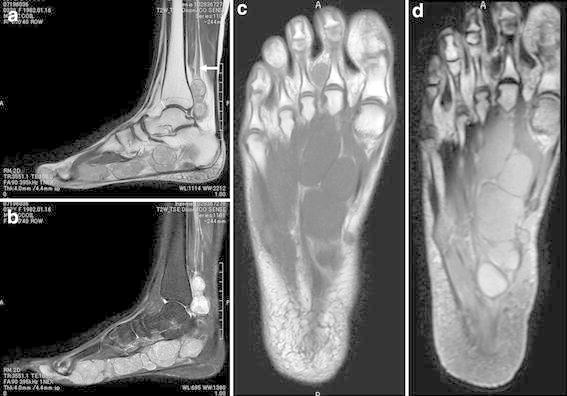
Magnetic resonance imaging of the right foot and ankle. **a** Sagittal T2-weighted image showing multiple hyperintense lesions along the course of the posterior tibial (*white arrow*) and medial plantar nerves. **b** Sagittal T2-weighted image with fat suppression demonstrating multiple nodular lesions with heterogeneous high signal intensity. **c** Coronal T1-weighted image showing the lesions with iso-signal intensity relative to adjacent muscle. **d** Coronal contrast-enhanced T1-weighted image with fat suppression revealing mild to moderate enhancement of the lesions.

A 32-year-old woman was referred to our hospital with a 5-year history of a slowly growing, painful mass in the medial aspect of the right ankle. There was no history of antecedent trauma. A family history of NF2 or schwannomatosis was not evident. The patient’s past medical history was unremarkable. On physical examination, an elastic-hard, poorly mobile, tender mass was noted in the medial aspect of the ankle (Figure 1), with extension into the medial plantar aspect of the foot. Tinel sign was elicited with radiation into the plantar aspect of the foot. Abnormal sensations including numbness and burning were present in the plantar aspect of the foot. Manual muscle testing revealed mild weakness (grade 4) in the right toe flexor muscles (I–IV). Laboratory data were within normal limits. Magnetic resonance imaging (MRI) demonstrated multiple nodular lesions with iso-signal intensity relative to skeletal muscle on T1-weighted sequences and heterogeneous high signal intensity on T2-weighted sequences. Contrast-enhanced fat-suppressed T1-weighted sequences showed mild to moderate enhancement (Figure 2). There was no evidence of osseous involvement. Based on these features, a multinodular/plexiform schwannoma was strongly suspected.

**Figure 3:**
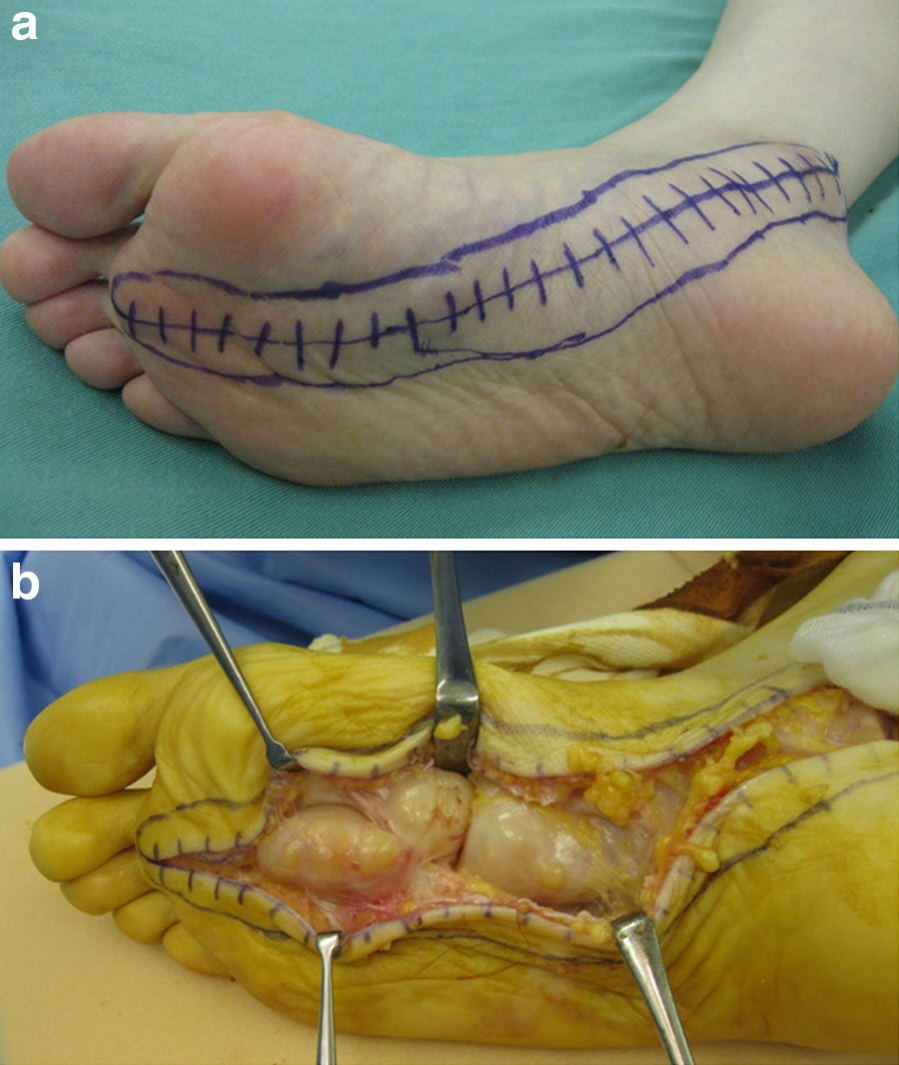
Intraoperative photographs. **a** The skin incision was made to provide adequate exposure of both nerve and tumor. **b** The tumor was observed to cohesively follow the course of the medial plantar nerve.

**Figure 4:**
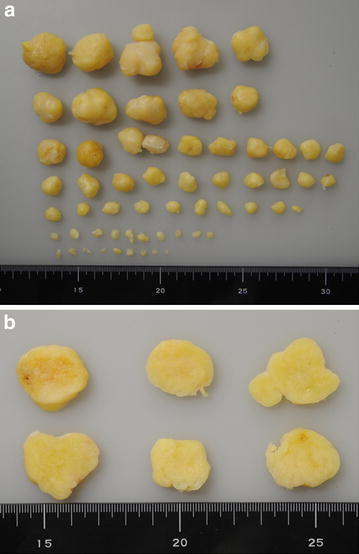
Gross findings. **a** Fifty-eight tumors which were enucleated; the largest was 3.0 cm in diameter. **b** Representative cut sections displaying *yellow-white* appearance.

**Figure 5:**
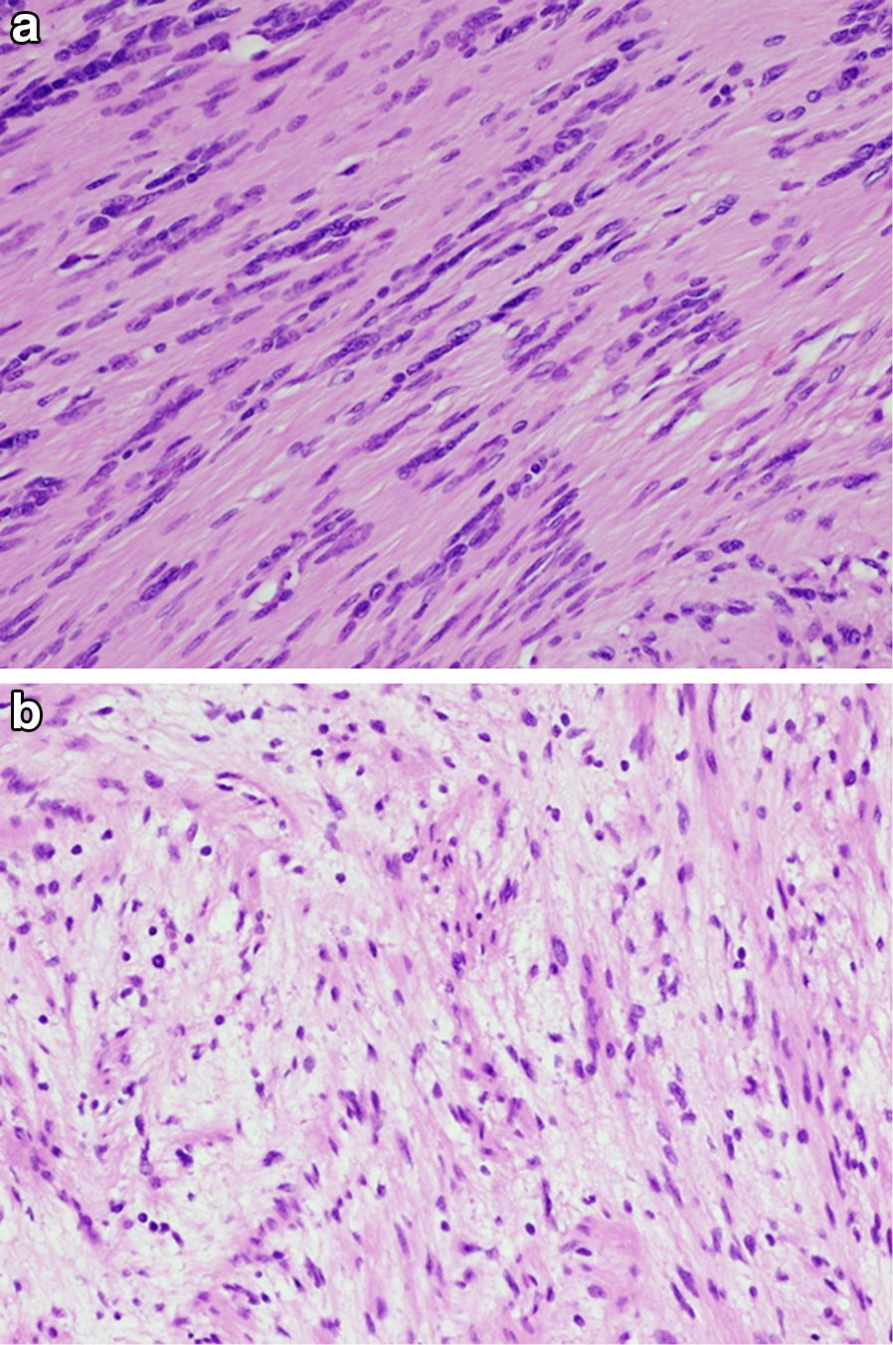
Histological findings. **a** Antoni A areas illustrating nuclear palisading. **b** Hypocellular Antoni B areas.

Following an open biopsy, the surgical enucleation was performed under general anesthesia with pneumatic tourniquet control and loupe magnification. The patient was placed in the supine position. The skin incision was made in the center over the tumor and extended along the course of the posterior tibial nerve and its larger terminal blanch (the medial plantar nerve) (Figure 3a). The tumor was observed to cohesively follow the course of the posterior tibial nerve through the tarsal tunnel, as well as the medial plantar nerve through the plantar aspect of the foot to the proximal phalanx (Figure 3b). We carefully made a longitudinal incision in the epineurium far away from the fascicles. The epineurial layers were gently peeled out until the shiny surface of the tumor was exposed. We did not carry out intraoperative nerve conduction studies to identify nonfunctioning fascicles. Fifty-eight separate tumors were shelled out in one piece. The dissected window of the epineurium was left open. The subcutaneous tissue was reapproximated using 4–0 absorbable sutures. Skin closure was achieved with 4–0 nonabsorbable sutures. A dry, sterile, minimally compressive dressing was applied to the patient’s foot and ankle. A short leg posterior splint was then applied. Macroscopically, individual tumors were yellowish-white and ranged from 0.2 to 3.0 cm in greatest diameter (Figure 4). Microscopically, all enucleated tumors showed a proliferation of spindle-shaped cells arranged in fascicles with occasional nuclear palisade arrangement in Antoni A areas (Figure 5a). Loosely arranged reticular portions (Antoni B areas) were also focally seen (Figure 5b). Neither highly cellular areas nor mitotic figures were found. These findings confirmed the diagnosis of multinodular/plexiform schwannoma.

**Figure 6:**
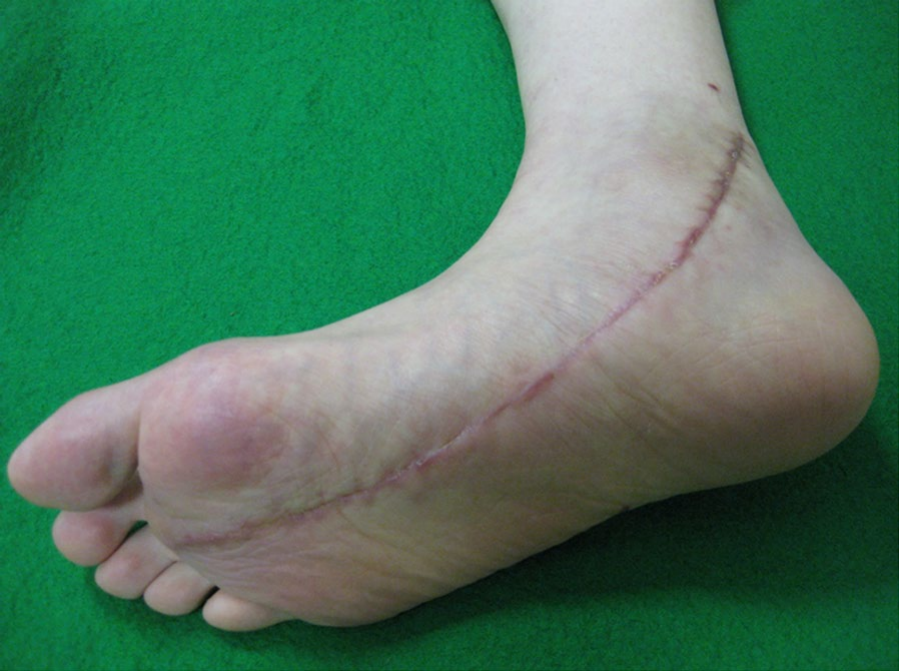
Clinical photograph of the surgical site at 2 months postoperatively.

The postoperative course was uneventful. The burning sensation was relieved immediately after surgery. The sutures were removed 2 weeks after surgery, by the same time the patient started progressive weight bearing. The patient then resumed full weight bearing activities within 6 weeks. At 5 months postoperatively, the patient had excellent pain relief and no aggravated neurological deficit except for mild hypoesthesia in the medial plantar aspect of the foot (Figure 6).

## Discussion and evaluation

Multinodular/plexiform schwannoma is a distinctive subtype of schwannoma, accounting for only 5% of all schwannomas. It develops predominantly in young adults with no gender predilection (Katsumi et al. 2003). Most multinodular/plexiform schwannomas are small with a greatest diameter less than 2 cm, originating from superficial nerves (Ikushima et al. 1999). Trauma might play an etiological role in the development of this tumor (Jacobson et al. 2011; Li et al. 2014). Histologically, the lesion can display a conventional, cellular, or mixed appearance (Agaram et al. 2005). Neurofilament protein immunoreactive axons are usually identified within the lesion (Rodriguez et al. 2012). The histological features of our case were similar to those of conventional schwannoma.

MRI is extremely useful in defining the extent of deep-seated multinodular/plexiform schwannoma (Hébert-Blouin et al. 2010; Katsumi et al. 2003; Mangrulkar et al. 2007). In addition, MRI seems to be the investigation of choice for identification of the nerve of origin. The lesion typically displays iso-signal intensity relative to skeletal muscle on T1-weighted images and high signal intensity on T2-weighted images, as in our case. A target sign may be observed on T2-weighted images (Hébert-Blouin et al. 2010). Internal curvilinear strands of low signal intensity on T1- and T2-weighted images represent thin fibrous septa between the individual nodules of the multinodular tumor and can be a valuable imaging feature of this condition (Ikushima et al. 1999; Jacobson et al. 2011). The pattern of enhancement is variable (Hébert-Blouin et al. 2010).

The decision to operate on the patients with benign neurogenic tumors should be based on the balance between the risk and benefit of the surgery. Several surgical techniques are attempted for solitary schwannomas arising from major peripheral nerves in the extremities (Date et al. 2012; Donner et al. 1994; Kim et al. 2012; Nishio et al. 2013). However, it would be difficult to completely resect the lesions without causing significant neurological deficits in larger multinodular/plexiform schwannomas. To minimize the risk of nerve injury, we performed the intracapsular enucleation under loupe magnification. With regard to the surgical enucleation, several key aspects of the operative technique are worthy of additional discussion and emphasis. Only a minimum longitudinal incision in the epineurium should be made to protect the remaining fascicle running over the epineurium. More importantly, great attention must be paid to meticulous dissection of the proximal and distal poles of the tumor. To achieve adequate exposure for the lesions located in the foot and ankle, it would be necessary to dissect the fibrous flexor retinaculum overlying the tarsal tunnel, as in our case. We believe that intracapsular enucleation is an effective and feasible method for preserving the neurological function.

The main differential diagnosis includes plexiform neurofibroma. Plexiform neurofibroma is one of the more commonly occurring plexiform neurogenic tumors and is essentially pathognomonic of neurofibromatosis type 1 (NF1). It can be present at birth or develop within the first year of life (Korf 1999). There is often nodularity and involvement of nerve branches that create the appearance of a serpentine “bag of worms”. Complete resection is typically not possible without the risk of major neurological compromise for the lesions affecting multiple nerve fascicles. The distinction between multinodular/plexiform schwannoma and plexiform neurofibroma is important, because plexiform neurofibroma has the potential for malignant transformation (Korf 1999). Previous studies have indicated that certain MRI features can be helpful for differentiating between these two lesions (Hébert-Blouin et al. 2010; Li et al. 2014). In our case, the imaging features were strongly in favor of multinodular/plexiform schwannoma and the definite diagnosis was confirmed by histological examination.

Limitations of this case study are lack of electrophysiological information and short postoperative follow-up.

## Conclusions

We have described the rare case of a large multinodular/plexiform schwannoma involving the posterior tibial and medial plantar nerves in a young adult woman. MRI is a clinically useful modality in the evaluation and detection of deep-seated multinodular/plexiform schwannoma. Intracapsular enucleation seems to be an acceptable treatment for this peculiar condition of the foot and ankle.

### **Consent**

Written informed consent was obtained from the patient for the publication of this report and any accompanying images.

## Abbreviations

MRI: Magnetic resonance imaging
NF1: neurofibromatosis type 1
NF2: neurofibromatosis type 2.
